# The effects of seasonal variations on household water security and burden of diarrheal diseases among under 5 children in an urban community, Southwest Nigeria

**DOI:** 10.1186/s12889-022-13701-z

**Published:** 2022-07-15

**Authors:** Patrick A. Akinyemi, Olusegun T. Afolabi, Olufemi O. Aluko

**Affiliations:** 1 Department of Community Health, Obafemi Awolowo University Teaching Hospitals Complex, Ile-Ife, Osun State Nigeria; 2 National Postgraduate Medical College of Nigeria, Ijanikin, Lagos State Nigeria; 3 Department of Community Health, Obafemi Awolowo University, Ile-Ife, Osun State Nigeria

**Keywords:** Seasonal variations, Household water security, Diarrhea disease, Southwest Nigeria

## Abstract

**Background:**

Household water security encompasses water-related factors that pose threats to public health at the household level. It presents a reliable access to water in sufficient quantity and quality towards meeting basic human needs. This study assessed the dynamics of seasonal variations in household water security and the association between household water security and diarrheal disease across dry and wet seasons in an urban settlement in Southwest Nigeria.

**Methods:**

A panel study design was employed to study 180 households selected using a multistage sampling technique. The selected households were studied during dry and rainy seasons. Household water security was assessed through the application of the all or none principle to 9 indicators associated with household water security. The intensity of water insecurity was also assessed using the nine indicators. The higher the number of indicators a household failed, the higher the intensity of household water insecurity. The association between the intensity of household water insecurity and the burden of diarrheal disease across the seasons was assessed using the Mantel-Haenszel test.

**Results:**

No household was water-secure in both dry and rainy seasons; however, the intensity of insecurity was more pronounced during the dry season compared with the rainy season. Ninety households (52.0%), had water insecurity intensity scores above fifty percentiles during the dry season while 21 (12.1%) households had a water insecurity score above the 50th percentile during raining season, *p* < 0.001. The burden of diarrheal disease was significantly higher among households with a water insecurity intensity score above the 50th percentile, 9 (8.1%) compared to households with a water insecurity intensity score below the 50th percentile 7 (3.0%), *p* = 0.034.

There was no statistically significant association between the intensity of water insecurity and diarrheal disease burden across the dry and rainy seasons, *p* = 0.218.

**Conclusion:**

The high burden of household water insecurity deserves concerted efforts from all concerned stakeholders, a panacea to an important health threat in the developing world.

**Supplementary Information:**

The online version contains supplementary material available at 10.1186/s12889-022-13701-z.

## Introduction

Household water security (HWS) encompasses households’ access to an adequate volume of potable water to meet their basic needs, incorporating various water-related factors that may threaten the health and livelihood of household members. Though there are several definitions of household water security from various water-related authorities, there are similarities in their components [1, 2]. These common components of household water security like water quality, per capita water consumption, access, reliability, resilience (seasonal variation in water supply services), and affordability indicators formed the basis for this study.

Household water insecurity has been shown to have numerous health effects which can be broadly referred to as water-related diseases. Though there is a dearth of studies that have assessed this concept in sub-Saharan Africa (SSA), a review of studies that have assessed water-related disease components independently showed that SSA is one of the worst-hit regions globally. The most prevalent among these diseases is diarrheal disease which is one of the leading childhood killer diseases in SSA. The prevalence of diarrheal diseases in Nigeria was 14.3% while it was 9.0% in Osun State [3].

Previous studies have demonstrated the possibility of seasonal variations in some components of household water security like accessibility and water quality [4–6]. The burden of diarrheal disease has also been shown to vary across seasons in some studies with a peak during the dry season [4, 6–8] while a bimodal pattern was observed in a study conducted among under-5 children in Ghana [9].

Previous post-disaster assessments of household water security as a risk factor for diarrheal disease demonstrated significant associations between household water security and diarrheal diseases [10, 11]. Most of the previous studies in Nigeria and SSA that seek to study water-related factors as risk factors for diarrheal disease have focused more on water quality [6, 12–15]. There are very few studies that assess other components of household water security as risk factors for diarrheal disease. This study therefore aimed at providing a comprehensive view of water-related factors that could potentially affect household water security and their association with diarrheal disease, putting into consideration the two major seasons in the study area.

Great effort has been expended on water insecurity indicators development and scale validation globally [16], hence, experiential scale-based and resource-based metrics, of diverse dimensions and applications [17, 18]. These depended on evidence-based secondary data architecture for classifications. In this study, a blended approach was utilized, based on primary data in randomly selected households, across wet and dry seasons. Guided by Jensen and Wu [17], Rosinger and Young [19], Liu et al. [16], and Octavianti and Staddon [18], the study considered the four primary domains of water insecurity: potable water availability, accessibility, use, and reliability, measured at microlevels. The composite water insecurity index focuses on adequacy (quantity available per person/day), accessibility, perception of quality, and reliability. However, the composite scale did not consider water hazard security and institutional related indicators, and therefore, could be expanded in future studies to encompass additional water insecurity domains.

Diarrhea is characterized by the passage of three or more loose or liquid stools per day while chronic diarrhoea denotes the passage of loose, watery stools that lasts for more than 2 weeks [20]. In Africa, diarrhea accounts for 7.7% of all deaths [21] while the national prevalence of diarrhea among under-5 children in Nigeria was 14.3% with a higher prevalence in the rural (15.0%) relative to urban settlements (12.6%) [22]. Each episode of diarrhea contributes to a significant nutritional deprivation which negatively affects child growth. Diarrhea has multiple aetiological agents and has been associated with domains of water insecurity [23, 24].

## Methods

This study was conducted at Ile-Ife, an ancient town situated in the tropical rainforest belt of Nigeria. The town covers an area of 1791 km^2^’, 7°28′N 4°34′E. The study area comprises two local government areas, namely Ife Central and Ife East Local Government Areas (LGAs). Both local governments are urban local government areas. The town has social amenities like electricity, primary and secondary educational facilities, and road networks. Public and private health care facilities are also evenly distributed across the town; each ward has at least a primary health care centre. The town also has a secondary health facility and hosts a unit of the Obafemi Awolowo University Teaching Hospital, a tertiary healthcare facility.

Ile-Ife, just like most other communities in the state, has no functional public water supply due to the infrastructural decay of existing public water supply systems. The residents of the town, therefore, rely mainly on private drinking water supply systems which makes water quality assessment in the study area challenging for concerned authorities. Furthermore, given that in a tropical rainforest that usually experiences heavy rainfall between March and November every year, rainwater harvesting is a major source of drinking water in the study area. Other natural sources of water, such as springs, ponds, and rivers are less commonly used because the community is urban, which should ordinarily be served by a conventional water treatment system; the natural sources are not readily available in the study area when compared with rural communities.

### Study design and study population

The study was conducted using a panel study design. This entailed having cross-sectional studies on household water security during dry and rainy seasons. The study population was households with under-five children in Ile-Ife, Osun State. This population was chosen because diarrheal disease is a water-borne disease of particular public health importance among under-5 children.

### Sample size and sampling technique

The sample size (n) was calculated to get an absolute precision of ±5% using the sample size formula for comparing two proportions [25].

$$n=\frac{2\left({Z}_a^2+{Z}_{\beta}^2\right) xp\left(1-p\right)}{d^2}$$

where Zα = Standard normal deviate corresponding to confidence level; at 99% level of confidence, Zα = 2.58 for a two-tailed test. Zβ = Standard normal deviate corresponding to power (1-β); at 90% power, Zβ = 1.64

P0 = proportion of drinking water source positive for the thermotolerant coliform count in the dry season, 20.5% = 0.205.

P1 = proportion of drinking water source positive for thermotolerant coliform count in rainy season, 42.3%, 0.423.

$$p=\frac{\left(0.205+0.423\right)}{2}=0.314$$

$$\mathrm{d}=0.423-0.205=0.21841$$

Thus,

$$\mathrm{n}\ \left(\mathrm{per}\ \mathrm{group}\right)=\frac{\ 2\left(2.58+1.64\right)2\times 0.314\ \left(1-0.314\right)}{(0.218)^2}$$

$$=\frac{35.62\times 0.22}{0.048}$$

$$=159.5\approx 160$$

$$\mathrm{Non}-\mathrm{Response}\ \mathrm{Rate}\ \left(\mathrm{NRR}\right)\ \mathrm{of}\ 10\%=\frac{n}{1- NRR}$$

$$=\frac{160}{1-0.1}$$

$$=177$$

This gives the minimum sample size of 177 households. This was rounded up to 180. The total sample size for the study was, therefore, 180 households.

After correcting for an attrition rate of 10%, the minimum sample size was 180 households. The sample size was calculated based on the proportions of drinking water sources positive for thermotolerant coliform counts during dry and rainy seasons in Port Harcourt Nigeria, 20.5 and 42.3% respectively [26].

A multistage sampling technique was adopted in enrolling the households. Ife Central local government area was selected out of the two local government areas in the town using a simple random sampling technique, balloting method. Five wards were subsequently selected out of 11 wards that made up the local government area using a simple random sampling technique, the balloting method. The sample size was equally distributed across the five selected wards. Households were selected using a systematic random sampling method, using an updated house listing from the most recent Immunization Plus Days as the sampling frame. Female heads of households were enrolled and interviewed. The same households selected were interviewed in both dry and rainy seasons.

### Data collection

The data were collected in December 2019 (dry season period) and June 2020 (rain season period) using a pretested, interviewer-administered questionnaire, with sections on the socio-demographic characteristics of households and water use patterns. The section that assessed household water security was structured based on the model developed by Thomas [27] and comprised 10 indicators: per capita water consumption, quality of water infrastructure, water quality, users’ satisfaction, the proximity of water sources, conflict or emotional distress while accessing water sources, reliability of the source, the resilience of water sources, collective management, and affordability of water for the households. Per capita, water consumption was assessed using a combination of water diary and container estimation.

The water diary contains basic household activities that require water use. Containers used to carry out the activities were sighted to avoid underestimation and exaggeration of water quantity consumed per day. The quantity estimated per activity was multiplied by the frequencies of the activities to obtain the estimated quantity consumed per day. A proforma for sanitary risk inspection of the source of water was adapted from the World Health Organisation [28]. The sanitary risk inspection tool contained 10 questions tailored to suit different sources of water for domestic consumption. The higher the score the poorer the sanitary status of the water source.

Research assistants with a tertiary level of education were employed for the study and underwent a two-day intensive training. The training involved ethical compliance, approaches to field data collection, contents of the field data collection instruments, and a practical demonstration of data collection tool usage: questionnaire, sanitary risk inspection proforma, and water diary. Administration of the tools was also practiced in selected households under supervision to ensure that the data collection method was uniform and followed the training received. Diarrheal disease was assessed using the WHO definition as the operational definition; passage of watery stool of more than three episodes per day in the last two weeks before the survey. A water diary was administered based on the activities of the respondents within the last one week before the survey to cover activities that are not being carried out daily. Containers for each activity were sighted by the research assistants to get an estimate of the volume of water being used per activity while the participants gave information about the frequency of the activities per day. The volume of water used for activities that are not carried out on daily basis was divided by the number of days in between performances of the activities to get an estimate of volume per day.

### Data analysis

Data were analysed using IBM SPSS version 25 for Windows. Categorical variables like sociodemographic characteristics, components of household water security, and seasonal variations in sources of water were summarized using frequencies and percentages. Continuous variables like the intensity of household water insecurity were summarized using median and interquartile range (IQR). Using multiple response analyses, water sources were presented using frequencies and percentages in the form of a composite bar chart.

Differences in the components of household water security across the dry and rainy seasons were assessed using the Chi-Square test while the difference in intensity of household water insecurity across the seasons was assessed using the Wilcoxon Sign Rank test. The intensity of household water insecurity was classified based on the number of indicators that the households recorded poor performance. Households were ranked into two groups based on the household water insecurity score; those with scores below the 50th percentile and those with the median score (50th percentile) and above. The Association between the intensity of household water insecurity and the burden of diarrheal disease was assessed using Chi-Square. The relationship between household water security and the burden of diarrheal diseases putting seasonal variation into consideration was assessed using the Mantel-Haenszel test.

A *p*-value of less than 0.05 was considered statistically significant.

## Results

One hundred and eighty households were recruited for the study; however, 173 households completed the second phase of the study giving a completion rate of 96.1%. The reason for the loss to follow-up was relocation from the houses where they were initially enrolled. The comparison between the rainy and dry seasons was based on 173 households that completed both arms of the study.

Table 1 shows the sociodemographic characteristics of the households enrolled in the study. Three-quarters (74.4%) of the households were Christians while one-quarter were of the Islamic faith. The secondary level of education was the highest level of education completed by most heads of households (134, 74.4%).

**Table 1:** Sociodemographic characteristics of the households

Variables	Frequency (%) ***N*** = 180
**Religion**	
Christianity	134 (74.4)
Islam	45 (25.0)
Other	1 (0.6)
**Marital Status**	
Married	175 (97.2)
Single never married	3 (1.7)
Widowed	2 (1.1)
Types of Marriage**	
Monogamous	160 (90.4)
Polygamous	17 (9.6)
**Tribe**	
Yoruba	158 (87.8)
Igbo	10 (5.5)
Hausa	9 (5.0)
Others	3 (1.7)
**Occupation**	
Trading	95 (52.8)
Artisan	54 (30.0)
Civil servant	13 (7.2)
Housewife	9 (5.0)
Farming	5 (2.8)
Others	4 (2.2)
**Level of Education of Female Head**	
No formal education	9 (5.0)
Primary	13 (7.2)
Secondary	134 (74.5)
Tertiary	24 (13.3)

** *N* = 177

**Fig. 1:**
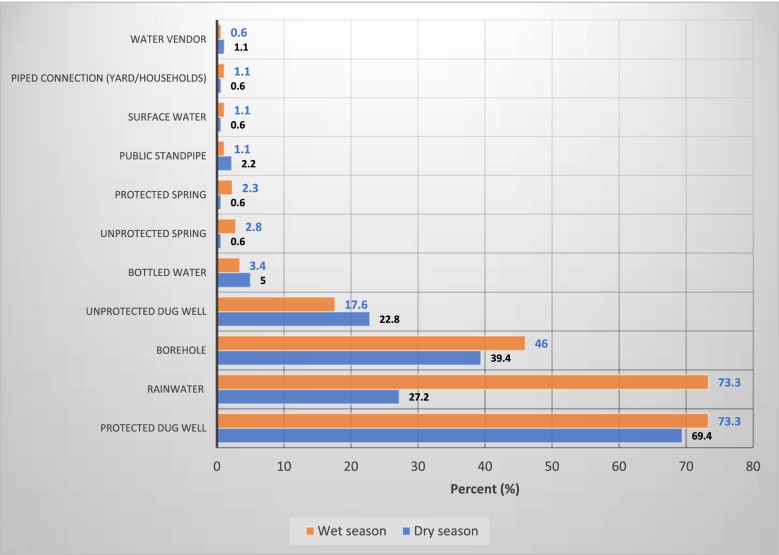
Common water sources for household use during dry and rainy seasons

During the rainy season, households used varying sources of water for drinking and domestic uses with protected dug wells and harvested rainwater accounting for 73% of sources while boreholes contributed 46% and unprotected dug well in less than one-fifth of the households, 17.6%. During the dry season, a protected dug well was the most common source of water for households (69.4%), followed by boreholes (39.4%). The use of rainwater was also observed in about 3 out of 10 households (27.2%) and 22.8% of households used unprotected dug well (Fig. 1).

**Fig. 2:**
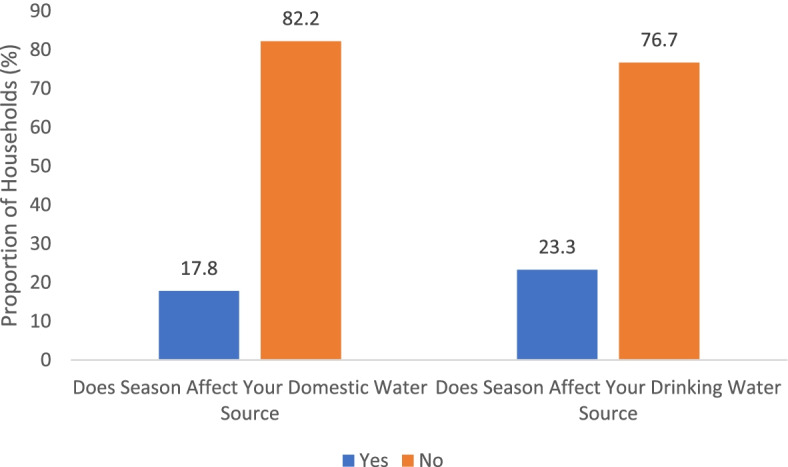
Experience of seasonal variation in household water sources

Over three-quarters of respondents reported that their sources of household water for drinking and domestic needs were affected by seasonal changes (Fig. 2).

Table 2 shows the components of household water security. About one-quarter, 44 (25.4%) of the households used less than 50 l per capita per day (l/c/d) during the dry season while 37 (21.4%) used less than 50 l/c/d during the rainy season. The risk of contamination at the water sources was high among 63 (36.4%) households during the dry season (63,36.4%) as well as during the rainy season (60,34.7%). No household made more than 30 minutes round trip to access water during the rainy season while almost half of the households, 84 (48.6%) experienced having more than 30 minutes round trip to access water during the dry season. Less than one-tenth, 13 (7.5%) of the households were not satisfied with the quality of their water during the rainy season while 65 (37.6%) of households were not satisfied during the dry season.

**Table 2:** The Components of Household Water Security

Description of Variables	Indicators	Dry Season N (%)	Rainy Season N (%)	Statistics
**Quantity**	**Per capita water consumption**			
	Less than 50 l/c/d	44 (25.4)	37 (21.4)	𝟀^2^ = 0.7898
	50 l/c/d and above	129 (74.6)	136 (78.6)	*p* = 0.374
**Quality**	**Quality of water source infrastructure (sanitary risk inspection score)**			
	Low risk	110 (63.6)	113 (65.3)	𝟀^2^ = 0.1135
	High risk	63 (36.4)	60 (34.7)	*p* = 0.736
	**Satisfaction with water quality**			
	Satisfied	108 (62.4)	160 (92.5)	𝟀^2^ = 44.7562
	Not satisfied	65 (37.6)	13 (7.5)	*p* < 0.001
Accessibility	**The proximity of water source**			
	Less than 30 minutes round trip	89 (51.4)	173 (100.0)	𝟀^2^ = 107.9764
	More than 30 minutes round trip	84 (48.6)	0 (0.0)	*p* < 0.001
	**Experience of conflict or emotional distress in accessing water**			
	No	159 (91.9)	66 (96.0)	𝟀^2^ = 0.1473
	Yes	14 (8.1)	7 (4.0)	*p* = 0.701
Reliability and resilience	**Reliability: experience of malfunctioned water source for more than 2 weeks**			
	No	159 (91.9)	168 (97.1)	𝟀^2^ = 4.5109
	Yes	14 (8.1)	5 (2.9)	*p* = 0.034
	**Resilience: Seasonal variation in drinking water source**			
	No	134 (77.5)	120 (69.4)	𝟀^2^ = 2.4726
	Yes	39 (22.5)	53 (30.6)	*p* = 0.1158
	**Collective management of water sources**			
	Good	140 (80.9)	160 (92.5)	𝟀^2^ = 10.029
	Poor	33 (19.1)	13 (7.5)	*p* = 0.002
Affordability	**Households spend more than 5% of their income on water**			
	No	172 (99.4)	173 (100.0)	𝟀^2^ = 0.000
	Yes	1 (0.6)	0 (0.0)	*p* = 0.997

The proportion of households that experienced conflicts or emotional distress while accessing water during the rainy season was 4.0% while those that had a similar experience during the dry season was 8.1%. Most of the households had reliable water sources in both rainy, 168 (97.1%), and dry seasons 159 (91.9%). Less than one-tenth, 13 (7.5%), of the households, experienced poor collective management of water resources during the rainy season compared to about one-fifth of the households 33 (19.1%), during the dry season. Only one household (0.6%) spent above 5 % (5%) of its monthly income on procuring water during the dry season. All households had access to affordable water during the rainy season.

**Fig. 3:**
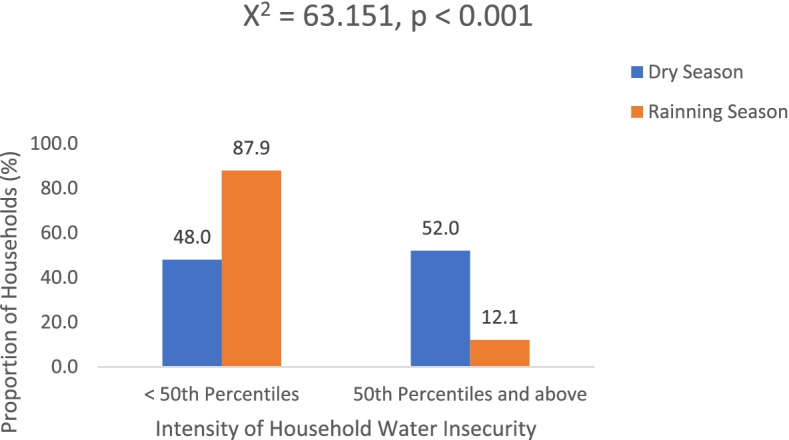
Intensity of water insecurity during dry and rainy season

The median intensity of water insecurity was significantly higher during the dry season, 3.0 (1.0) compared with the rainy season, 2.0 (1.0), *p* < 0.001. Based on the number of indicators that qualified the households to be water insecure, the intensity of water insecurity varied. Figure 3 showed that more than half of the households 90 (52.0%) had the intensity of water insecurity above 50th percentiles during the dry season while 21 (12.1%) households had a water insecurity score above the 50th percentile during raining season. The difference in the intensity of water insecurity experienced by households across dry and wet seasons was statistically significant, *p* < 0.001. The proportion of households with a water insecurity intensity score above the 50th percentile that experienced diarrheal disease, 9 (8.1%) was significantly higher than the proportion of households with a water insecurity intensity score below the 50th percentile 7 (3.0%), *p* = 0.034.

There was no significant difference between the prevalence of diarrheal disease and intensity of water insecurity across dry and rainy seasons, Mantel-Haenszel = 1.516, *p* = 0.218.

## Discussion

All the households that participated in this survey relied on private drinking water systems. This could be due to the non-functional status of public water supply systems which was attributed to decay in water supply infrastructure [29]. This is similar to the findings from a study conducted at Port Harcourt where less than 1% had access to public water supply [26]. This contrasts with significantly high proportions of households in studies conducted in Tanzania and South Africa which rely on public water supply [30, 31]. The differences observed in the systems of water supply could be due to variations in the study area with different water policies and varying degrees of implementation of the policies. Also, variation in the geographical locations of the studies with a subtle variation in weather conditions and access to water resources could have accounted for variation in major system of households’ water supply.

Protected dug wells were the main source of water in the study area in both dry and rainy seasons. The use of boreholes in both dry and rainy seasons was less common relative to the aforementioned water sources. This could be because boreholes are more expensive to construct compared to protected dug well, thus, limiting their availability. This was contrary to observations from a similar study conducted in Port Harcourt where the borehole was the major source of drinking water during the rainy season while packaged water (including sachet and bottled water) was the major source of drinking water during the dry season. The variations in the sources of water compared with this study could be a result of differences in the geographical factors being experienced in the study area; this study was conducted in a land-locked semi-urban area while the study at Port Harcourt was in a very urban, cosmopolitan oil-rich coastal city. The variation in the socio-economic status of the two study areas and underground water pollution, especially by crude oil, could account for the higher proportion of households that rely on packaged water in Port Harcourt compared with Ile-Ife.

More than one out of five households experience seasonal variation in their sources of drinking water. This could be due to rainwater harvesting being a major source of drinking water in the study area and its seasonal availability. Background information also showed that the majority of households use shallow wells with poor reliability. The rate of recharge of the underground water sources during dry seasons, which is the major source of water supply in the study area, may not meet the demand of households, hence, the variation in water sources across seasons. The proportion of respondents that experience seasonal variations was lower than that of respondents in Port Harcourt. This could be due to variations in the main sources of water in the study areas. The proportion that experiences seasonal variation in household water security in a study conducted in South Africa was very low (less than 1 out of 20 households) compared with more than 1 out of 5 households recorded in this study [30]. The difference could be due to more access to public water supply in South Africa compared to this study area where all households rely on the private drinking water system.

Based on the application of the all-or-none principle in scoring the components of household water security, no household was water secured in both dry and rainy seasons. The scoring was based on the knowledge of interconnectivity of the components of household water security in which a compromise in one of the components negatively affects others. Therefore, a failure of a household in one indicator renders the household water insecure. Rating households based on the number of indicators the households failed to meet in terms of intensity however showed that the intensity of water insecurity was more during the dry season.

The higher intensity of household water insecurity during the dry season was due to defects in varying components across households like reduction in water quantity in most shallow wells, and competition for good quality water from available boreholes which may not be easily accessible. The finding that no household was water-secure was in agreement with the findings from similar studies conducted in Bolivia and Bangladesh where virtually all respondents were water insecure [10, 11]. Assessment of the intensity of water insecurity among Bolivians showed that most adults that participated in the study had medium intensities of water insecurity while about one-quarter experienced the high intensity of water insecurity [11]. This is similar to the findings from the study where several households had an intensity score above the 50th percentile. This could be due to similarities in the status of infrastructures in the two study areas. The study among Bolivians was conducted post-disaster capable of destruction of existing infrastructure while the study population at Ile-Ife, though free from disaster over the study period, has experienced perennial social infrastructural decay. The study conducted among the Colonias at the USA-Mexico border however showed that more than 2 out of 5 households were water-secure [10]. The variation in findings could be a result of different methods of assessing and classifying household water security.

The proportion of households that reported diarrheal diseases was higher during the dry season than during the rainy season. The higher prevalence of diarrheal disease during the dry season was in agreement with previous studies conducted in varying socio-cultural settings [4, 6–8]. In another study, a bimodal distribution of the peak period of diarrheal disease was observed. It was reported that the burden of diarrheal disease peaked during the peak of rainfall and during the dry season around January [9]. The burden of diarrheal diseases was significantly affected by the intensity of water insecurity. A higher burden of diarrheal disease was observed in households with water insecurity intensity scores above 50th percentiles. This could be due to a higher risk of inadequate access to water for good hygiene practices or access to water with poor quality, thus increasing the risk of faeco-oral transmission of disease. There was however no significant association between household water security status and the prevalence of the diarrheal disease across the seasons. The insignificant association between the burden of diarrheal disease and the intensity of water insecurity across the seasons may be due to components of household water insecurity measures that are less affected by seasonal variation. Seasonal variations in the association between water insecurity and diarrheal disease were however not put into consideration in previous studies [10, 11].

## Limitations to the study

The assessment of diarrheal disease was self-reported and therefore prone to recall bias, multiple episodes within the reported period and misclassification due to wrong diagnosis. The reference period for the burden of diarrheal disease was however limited to two weeks before data collection in both seasons to limit the recall bias. The assessment of per capita water consumption using a water diary was also prone to recall bias or social desirability bias. To address this challenge, interviewers, requested to see the household water containers that were being used for specific domestic activities, particularly when the respondents didn’t know the volume or it was perceived to be exaggerated. The assessment of water quality was limited to the quality of water infrastructure (proneness of infrastructure to water contamination) and users’ satisfaction with their water’s physical quality. Findings from a few water samples that were collected randomly showed that all water samples had poor microbiological profiles. Findings from laboratory assessment of water quality may therefore not change the outcome of water security assessment, particularly because of the all-or-none principle applied to the indicators.

## Conclusion and recommendations for future study

Findings that no household was water-secure across both seasons is a serious concern. The intensity of water insecurity bites harder during the dry season, a season with a higher burden of diarrheal disease. Though the association between the intensity of water insecurity and the burden of diarrheal diseases across the seasons was not statistically significant, there may be a link between the two. There is therefore a need for both the government and private individuals to focus on all the components of household water security while designing and implementing water projects rather than focusing mainly on accessibility and to a less extent the quality of water.

Assessment of household water security in the rural and urban communities across the two main seasons is desirable as this will put into consideration the variation in socio-cultural environments. Assessment of household water security among households with access to public water supply systems could also be considered in the future study.

## Supplementary Information

**Additional file 1:** Household water security indicators [27].

**Additional file 2:** Questionnaire.

**Additional file 3:** Sanitary risk inspection/assessment.

**Additional file 4:** Water diary.

## Data Availability

The dataset for this study is not publicly available but it will be made available via communication with the corresponding author.
