# Supplementary figures and images for: The effects of seasonal variations on household water security and burden of diarrheal diseases among under 5 children in an urban community, Southwest Nigeria
Source: BMC Public Health. 2022 Jul 15;22:1354. doi: 10.1186/s12889-022-13701-z (PMC9284814; doi:10.1186/s12889-022-13701-z)

# HOUSEHOLD WATER SECURITY INDICATORS.


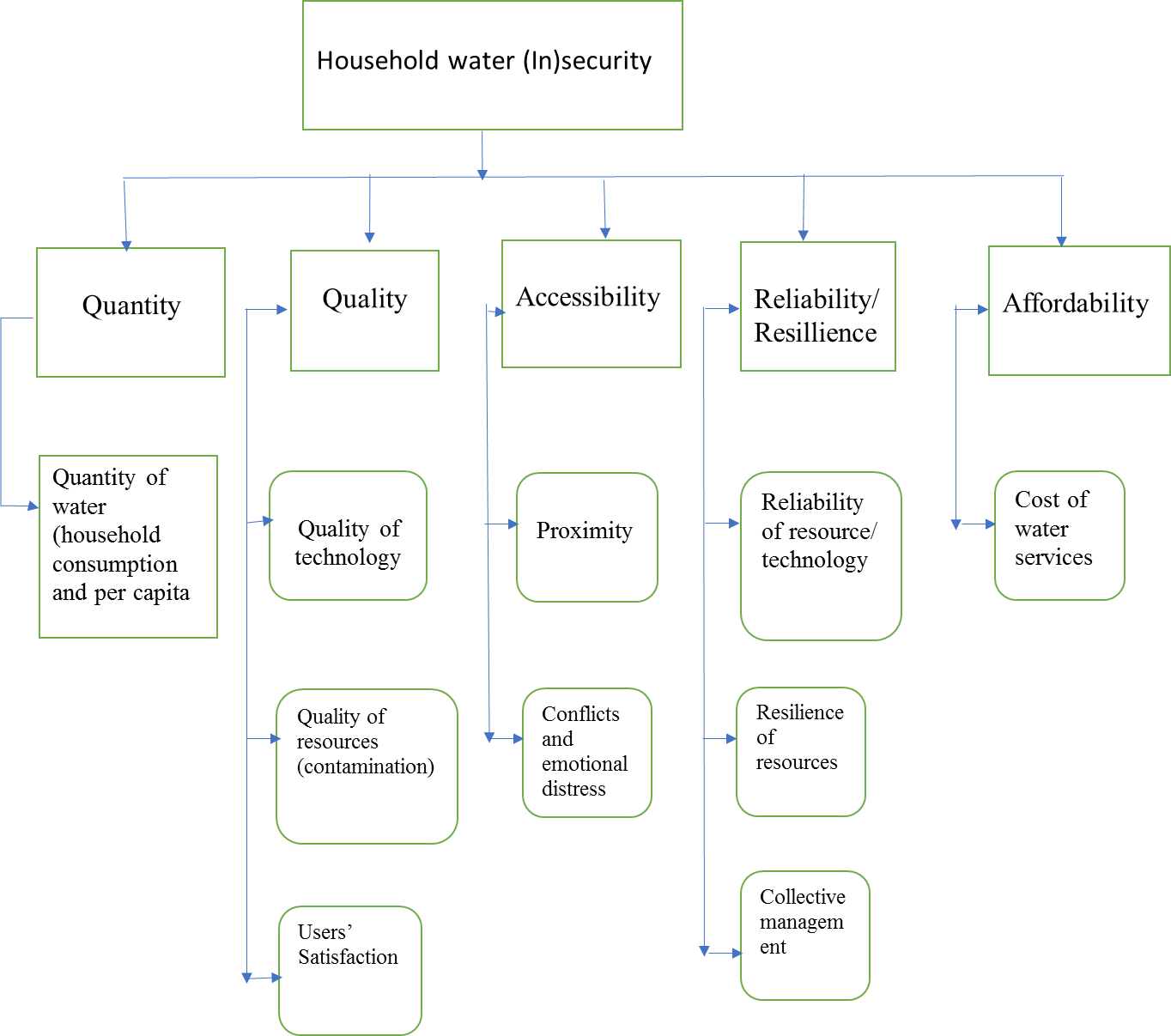


Source: Vincent Thomas, 2015[27](#_ENREF_48)

Supplement: Supplementary file 1 — Additional file 1: Household water security indicators [27]. [file 12889_2022_13701_MOESM1_ESM.docx]
