# Supplementary material for: The effects of seasonal variations on household water security and burden of diarrheal diseases among under 5 children in an urban community, Southwest Nigeria
Source: BMC Public Health. 2022 Jul 15;22:1354. doi: 10.1186/s12889-022-13701-z (PMC9284814; doi:10.1186/s12889-022-13701-z)
Supplement: Supplementary file 2 — Additional file 2: Questionnaire. [file 12889_2022_13701_MOESM2_ESM.docx]

# QUESTIONNAIRE

Good morning / good afternoon. My name is _____________________. I am part of a team of people who are assessing water practices in your community. Our team will interview approximately 100 households in this area. Your local leaders have granted us permission to conduct this study, and your house has been randomly selected to participate. If you agree to participate, I will ask you questions about your drinking-water and collect a sample of your water. The interview will take approximately 30 minutes. Information provided will be kept confidential. Would you like to participate?

Person obtaining consent Household number­_____________________

A. Interviewer _________________________________________

B Date _______________________________

C Time _______________________________

D Location ___________________________

1. Respondents Socio-Economic and Demographic Characteristics

| S/N | Questions and Options (Interviewer should circle the appropriate option. Interviewer should not write in the shaded portion) |
| --- | --- |
|  |  |
| 1 | Age   1. Female head of the household 2. Male head of the household |
| 2 | Religion  1 = Islam 2= Christianity 3=Others (specify} |
| 3 | Marital status  1 = Single Never married 2= Married 3= Separated or Divorced 4= Widowed 5=Others (specify) |
| 4 | If married, type of marriage  1=Monogamous 2= Polygamous |
| 5 | What is your tribe?  1= Yoruba 2 = Hausa 3= Igbo 4=Others (specify) |
| 6 | Occupation  0= housewife 1= trading 2= Artisan 3= Civil Servant (please specify____________________________)  4= farming  5= others (please specify______________________) |
| 7 | . Level of Education (Completed)  0= None 1= Primary 2= Secondary 3= Diploma 4=University |
| 8 | Level of Education of the male head of the household  0= None 1= Primary 2= Secondary 3= Diploma 4=University |
| 9 | Average monthly earnings for the household  ______________________________________ |
| 10 | Total number of people per household____________  Number of members by age group   \| Age group (years) \| Male \| Female \| Total \| \| --- \| --- \| --- \| --- \| \| Under-5 \|  \|  \|  \| \| 5-9 \|  \|  \|  \| \| 10- 19 \|  \|  \|  \| \| 20 and above \|  \|  \|  \| |
| 11. | House ownership status of a family  1= Rented from private owners 2= Personal own house 3= Joint ownership  4= Government property Others specify__________ |
| 12 | House type  1= single room, 2= a room and a Parlour, 3= two bedrooms flat, 4= others specify |

1. Structure and Properties in the House

| S/N | Questions and Options (Interviewer should circle the appropriate option. Interviewer should not write in the shaded portion) |
| --- | --- |
|  |  |
| 1 | Types of wall (OBSERVE)  1= Concrete 2= Cloth/Tarpaulin 3=Wood 4= Dirt 5= Metal  6= others (Please Specify___________________) |
| 2 | Type of floor (OBSERVE)  1= Concrete 2= Cloth/Tarpaulin 3=Wood 4= Dirt 5= Metal  6= others (Please Specify___________________) |
| 3 | Type of roof (OBSERVE)  1= Concrete 2= Cloth/Tarpaulin 3=Wood 4= Dirt/grass  5= Metal 6= others (Please Specify___________________) |
| 4 | How many of the following household Items does the respondent’s household owns  Bed_____, Bicycle_______, Motorcycle______, Car_____Radio______ Television______, Mobile Phone_______ Refrigerator______ |
| 5 | How many of the following household pets do you own?  Chicken ____, Goat____ sheep____ cattle______ Other (specify_____________) ______ |
| 6 | Number of rooms for sleeping |
| 7 | Means of sewage (excreta) disposal  1= Water closet 2= pit latrine 3= VIP latrine, 4= public toilet 5= open defecation  6= others (specify_________________) |
| 8 | Means of refuse (solid waste) disposal  1= open dumping, 2= burning 3= Burying 4= Refuse collector  5= others (specify______________________________) |
| 9 |  |

1. Water Demand and Use Patterns of Household

| S/N | Questions and Options (Interviewer should circle the appropriate option. Interviewer should not write in the shaded portion) |
| --- | --- |
|  |  |
| 1 | Sources of water for household use **(multiple response)**  1= Piped connection to yard or in household  2= Public standpipe 3= Borehole 4= Protected dug well 5= Protected spring 6=Rainwater 7= Unprotected dug well 8= Unprotected spring 9= Vendor water 10= Bottled water  11= Tanker 12= surface water sources 13. Others (specify) |
| 2 | Does change in season (dry and rainy season) affect your source of water for household use other than drinking?  0= No 1= Yes |
| 3 | If question 2 is Yes, which source of water do you use during raining season? ***List in order of frequency with most often used first***   1. ___________________________ 2. ___________________________ 3. ___________________________ |
| 4 | If question 2 is Yes, which source of water do you use during dry season? ***List in order of frequency with most often used first***   1. ___________________________ 2. ___________________________ 3. ___________________________ |
| 5 | Sources of drinking water **(multiple response)**  1= Piped connection to yard or in household  2= Public standpipe 3= Borehole 4= Protected dug well 5= Protected spring 6=Rainwater 7= Unprotected dug well 8= Unprotected spring 9= Vendor water 10= Bottled water  11= Tanker 12= surface water sources 13. Others (Specify) |
| 6 | Does change in season (dry and rainy season) affect your source of drinking water?  0= No 1= Yes |
| 7 | If question 6 is Yes, which source of drinking water do you use during raining season? ***List in order of frequency with most often used first***   1. ___________________________ 2. ___________________________ 3. ___________________________ |
| 8 | If question 6 is Yes, which source of drinking water do you use during dry season? ***List in order of frequency with most often used first***   1. ___________________________ 2. ___________________________ 3. ___________________________ |
| 9 | How often do you use the under listed sources of water?  1= daily 2= alternate day to weekly 3= monthly 4= more than monthly interval 5= not used at all   1. Pipe borne water [] 2. Borehole [ ] 3. Hand dug well [ ] 4. River [ ] 5. Stream [ ] 6. Lake [ ] 7. Pond [ ] 8. Rainwater [ ] |
| 10 | Which container do you use to collect water?  1= daily 2= alternate day to weekly 3= monthly 4= more than monthly interval 5= not used at all **(multiple responses allowed)**   1. Open bucket [ ] 2. Bucket with lid [ ] 3. Jerry can [ ] 4. Clay pot [ ] 5. Plastic pot [ ] 6. Basin [ ] |
| 11 | Which container do you use to store water?  a. Open bucket [ ]  b. Bucket with lid [ ]  c. Jerry can [ ]  d. Clay pot [ ]  e. Rubber pot [ ]  f. Basin [ ]  g. Drum (with cover) [ ]  h. Drum (without cover) [ ] |
| 12 | If the source of water is not within the household  **Time taken to collect water from outside:**  By Women   1. No. of Trips: _______________ 2. Time Taken Per Trip: __________ 3. Qty. of water collected ___________   By Men   1. No. of Trips: _______________ 2. Time Taken Per Trip: __________ 3. Qty. of water collected ___________   By Children   1. No. of Trips: _______________ 2. Time Taken Per Trip: __________ 3. Qty. of water collected ___________ |
| 13 | Mention the average distance of drinking water source from your home __________________________ |
|  | If response to question 1, 5 or 9 include pipe borne water answer question 14 & 15 |
| 14 | Do you have storage tank?  0= no 1= yes  If yes, what is the capacity_________ litres |
| 15 | Do you have any of the following water using fixtures   1. Bath showers [ ] 2. Flush toilet [ ] 3. kitchen with complete water supply serviced [ ] 4. Others specify,_____________________________ |
| 16 | If you use source of water other than pipe 0= no 1= yes  what is the main reason for doing so? **(multiple response)**  (a) High price of water [ ] (b) physical distance of tap water point from your home [ ]  (c) I Lack of access to tap water [ ] (d) high interruption of tap water supply [ ]  others,__________________________________ |
| 17 | For what purpose do you use water at home? **(multiple response)**  1= daily 2= alternate day to weekly 3= monthly 4= more than monthly interval 5= not used at all  (a)cooking [ ] (b) bathing [ ] (c) cleaning [ ] (d) flushing toilet [ ] (e) washing cloths [ ]  (f) drinking [ ] (g) all of the above [ ] (h) others specify ___________________________ |

1. Perception of water security

| s/no | Variables | Yes | No |
| --- | --- | --- | --- |
|  | **Access** |  |  |
| 1 | Are you comfortable with time required to walk from home to water source? Pls state average time required_______________ |  |  |
| 2 | Are you comfortable with the waiting time in line at the source? Pls state average time required_______________ |  |  |
|  | **Adequacy** |  |  |
| 3 | Do you worry over adequacy of water supply for your household? |  |  |
| 4 | Do you have to reduce the quantity of water used for various purposes? |  |  |
| 5 | Do you have to go all day without drinking? |  |  |
| 6 | Do you have to go to bed thirsty? |  |  |
|  | **Safety** |  |  |
| 7 | Dominant source of drinking water  Protected [ ]  Unprotected [ ]  Please mention the source____________________________ |  |  |
| 8 | Do you feel the source of water is undesirable/dirty? |  |  |
| 9 | Perception of having drunk dirty water |  |  |
|  | **Lifestyle** |  |  |
| 10 | Do you have to forego your daily activities like going to work, school or other places you considered important in order to go and fetch water? |  |  |

1. Water insecurity questions

| s/no |  |
| --- | --- |
|  | **Users’ Satisfaction with the water quality** |
|  | Do you feel the water you use is free of germs that can cause diseases? 0= No 1= yes |
|  | Are you satisfied with the following?   - 1. Appearance of the water? 0= No 1= yes   2. Taste of the water? 0= No 1= yes   3. Odour of the water? 0= No 1= yes |
|  | **Conflict and emotional distress**  In the last 2 weeks: |
|  | Have you gotten angry with someone about getting access to drinking water? 0= No 1= yes |
|  | Argue with someone? 0= No 1= yes |
|  | Felt worried about accessing drinking water? 0= No 1= yes |
|  | Afraid about access to drinking water? 0= No 1= yes |
|  | **Reliability and resilience** |
|  | Was your main water source dysfunctional (faulty) for a period of more than 2 weeks in the last one year? 0= No 1= yes |
|  | Have you been affected negatively by a reduction in the quantity of water due to change in seasons in the last one year? 0= No 1= yes |
|  | Have you been affected negatively by a reduction in the quality of water due to change in seasons in the last one year? 0= No 1= yes |
|  | **Collective management** |
|  | Do you have access to professionals (e.g. Plumber) in maintenance and repair of water supply system? 0= No 1= yes |
|  | Do you pay fee to any committee for the maintenance of water supply system (for public or community water supply only)? 0= No 1= yes |
|  | **Affordability** |
|  | How much do you pay for operation/ maintenance of your water source per month ­­­­­­­­­­______________ |

Prevalence of Diarrhoea Diseases

|  |  | 1st case  Age __________ | 2nd case  Age _________ | 3rd case  Age _______ |
| --- | --- | --- | --- | --- |
| 1. | Has (Name) had diarrhoea in the last 2 weeks?  (passage of 3 or more loose stool in one day) | Yes [ ]  No [ ]  Don’t Know [ ] | Yes [ ]  No [ ]  Don’t Know [ ] | Yes [ ]  No [ ]  Don’t Know [ ] |
| 2. | Was there blood in the stool? | Yes [ ]  No [ ]  Don’t Know [ ] | Yes [ ]  No [ ]  Don’t Know [ ] | Yes [ ]  No [ ]  Don’t Know [ ] |
| 3. | Was there mucous in the stool? | Yes [ ]  No [ ]  Don’t Know [ ] | Yes [ ]  No [ ]  Don’t Know [ ] | Yes [ ]  No [ ]  Don’t Know [ ] |
| 4. | Where did you seek care for the diarrhoea disease? | Public hospital [ ]  Private hospital [ ]  Pharmacy/chemist [ ]  Faith based clinic [ ]  Home care [ ]  Others [ ]specify ______________ | Public hospital [ ]  Private hospital [ ]  Pharmacy/chemist []  Faith based clinic []  Home care [ ]  Others [] specify ______________ | Public hospital [ ]  Private hospital [ ]  Pharmacy/chemist [ ]  Faith based clinic [ ]  Home care [ ]  Others [ ]specify ______________ |
| 5 | How was the diarrhea disease managed?  ***Multiple response*** | Intake of more than usual amount of fluid [ ]  ORS [ ]  SSS [ ]  Use of other home based fluid [ ]  Antibiotics [ ]  Give more than usual to eat [ ]  Continue breastfeeding [ ]  None of the above [ ]  Others [ ] specify ______________ | Intake of more than usual amount of fluid [ ]  ORS [ ]  SSS [ ]  Use of other home based fluid [ ]  Antibiotics [ ]  Give more than usual to eat [ ]  Continue breastfeeding [ ]  None of the above [ ]  Others [ ] specify ______________ | Intake of more than usual amount of fluid [ ]  ORS [ ]  SSS [ ]  Use of other home based fluid [ ]  Antibiotics [ ]  Give more than usual to eat [ ]  Continue breastfeeding [ ]  None of the above [ ]  Others [ ] specify ______________ |
| 6 | What is the cause of diarrhea disease? ***Multiple response*** | Dirty food [ ]  Dirty water [ ]  Exposure to someone with diarrhoea [ ]  Development of teeth [ ]  Others [ ] specify ________________  Don’t know [ ] | Dirty food [ ]  Dirty water [ ]  Exposure to someone with diarrhoea [ ]  Development of teeth [ ]  Others [ ] specify ________________  Don’t know [ ] | Dirty food [ ]  Dirty water [ ]  Exposure to someone with diarrhoea [ ]  Development of teeth [ ]  Others [ ] specify ________________  Don’t know [ ] |
