# Supplementary material for: The effects of seasonal variations on household water security and burden of diarrheal diseases among under 5 children in an urban community, Southwest Nigeria
Source: BMC Public Health. 2022 Jul 15;22:1354. doi: 10.1186/s12889-022-13701-z (PMC9284814; doi:10.1186/s12889-022-13701-z)
Supplement: Supplementary file 3 — Additional file 3: Sanitary risk inspection/assessment. [file 12889_2022_13701_MOESM3_ESM.docx]

# SANITARY RISK INSPECTION/ASSESSMENT

Site name/ nr___________ date/time_________________

| S/no | Sources of water and characteristics  0= no 1= yes | Q S/N | Code |
| --- | --- | --- | --- |
|  | PIPED WATER WITH SUPPLY TANKS: DISTRIBUTION SYSTEM |  |  |
| 1 | Do any taps or pipes leak at the sample site? |  |  |
| 2 | Does water collect around the sample site? |  |  |
| 3 | Is the area around the tap unsanitary? |  |  |
| 4 | Is there a sewer or latrine within 30m of any tap? |  |  |
| 5 | Has there been discontinuity in the last 10 days? |  |  |
| 6 | Is the supply main pipeline exposed in the sampling area?? |  |  |
| 7 | Do users report any pipe breaks within the last week? |  |  |
| 8 | Is the supply tank cracked or leaking? |  |  |
| 9 | Are the vents and covers on the tank damaged or open? |  |  |
| 10 | Is the inspection cover or concrete around the cover damaged or corroded? |  |  |
|  | **Total Score of Risks …….…./10** |  |  |
|  | **DEEP BOREHOLE WITH MECHANISED PUMPING** |  |  |
| 1 | Is there a latrine or sewer within 100m of the pumping mechanism? |  |  |
| 2 | Is there a latrine within 10m of the borehole? |  |  |
| 3 | Is there any source of other pollution within 50m (e.g. animal breeding, cultivation, roads, industry water-Related)? |  |  |
| 4 | Is there an uncapped well within 100m? |  |  |
| 5 | Is the drainage channel cracked, broken or needing cleaning? |  |  |
| 6 | Can animals come within 50m of the borehole? |  |  |
| 7 | Is the base of the pumping mechanism permeable to water? |  |  |
| 8 | Does water form pools within 2m of the pumping mechanism? |  |  |
| 9 | Is the well seal unsanitary? |  |  |
| 10 | Is the borehole cap cracked? |  |  |
|  | Total Score of Risks.…………. /10 |  |  |
|  | **BOREHOLE WITH HANDPUMP OR WELL** |  |  |
| 1 | Is there a latrine within 10m of the borehole? |  |  |
| 2 | Is there a latrine uphill of the borehole? |  |  |
| 3 | Are there any other sources of pollution within 10m of borehole? (e.g. animal breeding, cultivation, roads, industry etc) |  |  |
| 4 | Is the drainage faulty allowing ponding within 2m of the borehole? |  |  |
| 5 | Is the drainage channel cracked, broken or need cleaning? |  |  |
| 6 | Can animals come within 10m of the borehole? |  |  |
| 7 | Is the apron less than 2m in diameter? |  |  |
| 8 | Does spilled water collect in the apron area? |  |  |
| 9 | Is the apron or pump cover cracked or damaged? Y/N |  |  |
| 10 | Is the hand pump loose at the point of attachment? Or (for rope-washer pump is the pump cover missing) |  |  |
|  | Total Score of Risks …….…. /10 |  |  |
|  | **HOUSEHOLD PIPED WATER** |  |  |
| 1 | Is the tap sited outside the house (e.g. in the yard)? |  |  |
| 2 | Is the water stored in a container inside the house? |  |  |
| 3 | Are any taps leaking or damaged? |  |  |
| 4 | Are any taps shared with other households? |  |  |
| 5 | Is the area around the tap unsanitary? |  |  |
| 6 | Are there any leaks in the household pipes? |  |  |
| 7 | Do animals have access to the area around the pipe? |  |  |
| 8 | Have users reported pipe breaks in the last week? |  |  |
| 9 | Has there been discontinuity in water supply in the last 10 days? |  |  |
| 10 | Is the water obtained from more than one source? |  |  |
|  | Total score of risk ……………………. /10 |  |  |

Source: World Health Organisation, 2017^[28](#_ENREF_139)^
