# Supplementary material for: The effects of seasonal variations on household water security and burden of diarrheal diseases among under 5 children in an urban community, Southwest Nigeria
Source: BMC Public Health. 2022 Jul 15;22:1354. doi: 10.1186/s12889-022-13701-z (PMC9284814; doi:10.1186/s12889-022-13701-z)
Supplement: Supplementary file 4 — Additional file 4: Water diary. [file 12889_2022_13701_MOESM4_ESM.docx]

# WATER DIARY

| Activity | Amount of water used (L).  (***Estimated by research assistant)*** | Frequency of activity per day | Total water used in Litres |
| --- | --- | --- | --- |
| Brushing teeth (tap switched off/without tap) |  |  |  |
| Washing hands and face |  |  |  |
| Bath |  |  |  |
| Flushing toilet |  |  |  |
| Washing clothes (washing machine) |  |  |  |
| Washing clothes by hand |  |  |  |
| Washing dish in the sink |  |  |  |
| Drinking water |  |  |  |
| Others______________  ___________________ |  |  |  |
| Total amount of water used in a day |  |  |  |

Do you carry out any of these activities outside your household water supply? Yes [ ] No [ ]

If yes, please specify ________________________________________________________

Estimate the quantity of water required__________________________________________
